# Type 2 Diabetes and Cardiovascular Risk in Women

**DOI:** 10.1155/2015/832484

**Published:** 2015-03-26

**Authors:** Giuseppina T. Russo, Giovannella Baggio, Maria Chiara Rossi, Alexandra Kautzky-Willer

**Affiliations:** ^1^Dipartimento di Medicina Clinica e Sperimentale, Policlinico Universitario “G. Martino”, Via C. Valeria, 98121 Messina, Italy; ^2^Chair of the Gender Medicine, University of Padua, Via Giustiniani 2, 35128 Padua, Italy; ^3^Laboratory of Clinical Epidemiology of Diabetes and Chronic Diseases, Fondazione Mario Negri Sud, Via Nazionale 8/A, 66030 Santa Maria Imbaro, Italy; ^4^Gender Medicine Unit, Division of Endocrinology and Metabolism, Department of Internal Medicine III, Medical University of Vienna, Währinger Gürtel 18-20, 1090 Vienna, Austria

Cardiovascular diseases (CVD) are the leading cause of death, also in diabetic women. Since 1998, when Haffner et al. [1] stated that subjects with type 2 diabetes mellitus (T2DM) had a CVD risk “equivalent” to previous myocardial infarction, a large number of studies have shown that this relative risk for CVD due to diabetes is greater in women than in men [2].

CVD in diabetic subjects is not entirely related to chronic hyperglycaemia and a number of other factors such as dyslipidemia, hypertension, hormonal, genetic, and environmental factors, as well as low-grade systemic inflammation and endothelial damage, lifestyle behaviours, adherence to therapies, and/or psychosocial factors may contribute to the worst outcomes observed in diabetic women. Notably, it is increasingly recognized that many of these factors show gender differences in their prevalence and/or association with CVD events, and this aspect should be specifically targeted when aiming at primary or secondary CVD prevention in diabetic subjects.

In this special issue, we looked at CVD in women with diabetes from different perspectives, giving a great contribution to this topic, in terms of mortality, management of risk factors, and therapies.

Two papers of this special issue looked at sex differences in CVD mortality associated with diabetes. One conducted on a large population-based sample from Italy demonstrated an excess of mortality in diabetic subjects as compared to nondiabetic ones and a greater impact of diabetes in females than in males for mortality for all causes, for CVD, and for myocardial infarction and renal causes. In the other study, G. Luo et al. showed in a retrospective analysis that fasting plasma glucose was an independent predictor of in-hospital mortality for nondiabetic female patients.

Gender-specific prevalence and management of major and emerging CVD risk factors in different populations were also the main topic of several papers of this special issue.

The paper by S. Chen et al., with a very interesting experimental protocol, clarified the relationships of albuminuria, a well-recognized CVD risk factor, with circulating levels of angiopoietin-1 (Ang-1), Ang-2, and vascular endothelial growth factor (VEGF) in serum and urine.

Potential gender differences in the distribution and control of major CVD risk factors were investigated in another three very large high-risk populations. Thus, in the eControl Study, a study on 286,791 patients with T2DM in Catalonia, Spain, J. Franch-Nadal et al. found that cardiometabolic control was worse in subjects with prior CVD; but control of several risk factors showed gender differences, favouring women with prior CVD only for smoking and BP, whereas LDL-cholesterol (LDL-C) levels were remarkably uncontrolled in women both with and without CVD.

The results of an overall bad control of LDL-C in women were also demonstrated in a very large Italian diabetic outpatient population from the Annals Study Initiative. This study, conducted on 415.294 patients (45.3% women) from 236 diabetes outpatient centers in Italy, demonstrated that LDL-C management was worst in women with T2DM, who were monitored and reached targets less frequently than men and, similar to men, did not receive medications despite high LDL-C levels. These gender discrepancies increased with age and diabetes duration, exposing older women to higher CHD risk.

In accordance with the previously cited studies, also the BARI 2D Trial, in a very high-risk population of T2DM subjects with established coronary artery disease, found that although women were as aggressively treated with drugs as men, they less frequently met targets for HbA1c and LDL-C, suggesting potential sex differences in response to drug therapies used to treat diabetes, hypertension, and hyperlipidemia.

Beyond LDL-C, different papers of this special issue focused on atherogenic dyslipidemia, that is, the presence of low HDL-cholesterol (HDL-C), and high triglycerides, which may have an impact on CVD especially in diabetic women [3, 4]. K. Song et al. demonstrated, in an* in vitro* study on primary hepatocytes of SR-BI knockout (SR-BI−/−) mice, that ATPase-B1 is involved in HDL endocytosis and it may be a potential target for regulating HDL metabolism.

Diabetic women have been demonstrated to have a less atheroprotective HDL subpopulation profile when compared to nondiabetic ones [5]. Another study of the special issue examining HDL subpopulations distribution in that same population of CHD-free diabetic and control women showed an inverse relationship between markers of systemic inflammation and the more atheroprotective large *α*-1, *α*-2, and pre*β*-1 HDL subclasses, especially in diabetic women, suggesting that different HDL particles could affect the atherosclerotic process through the modulation of subclinical inflammation. A third paper on atherogenic dyslipidemia by J. He et al. demonstrated gender differences on the discriminatory power of triglyceride (TG) and the triglyceride to high-density lipoprotein-cholesterol ratio (TG/HDL-C) for insulin resistance in normoglycaemic Chinese subjects.

Furthermore, in normoglycaemic women with previous GDM, Y. Winhofer et al. demonstrated that despite the diagnosis of normal glucose tolerance after delivery, prior GDM women are characterized by persisting subtle glucose abnormalities and insulin resistance, decreased adiponectin, and increased CRP concentrations, thus exposing them to an increased CVD risk. Thus women with a history of GDM should be regarded as high-risk group for development of overt diabetes and CVD and special attention should be given to preventive measures among these females.

Another two articles of this special issue also examined the role of estrogen replacement therapy (ERT) or flavonoids on CVD risk factors. Thus, M. Boukhris et al. reviewed the controversial role of ERT on CHD in postmenopausal type 2 diabetic women, trying to define the place of ERT on CHD prevention, whereas R. D'Anna et al. investigated the effects of a 6-month treatment of polyphenols, myo-inositol, and soy isoflavones on several CVD risk factors in postmenopausal women with metabolic syndrome.

Finally, in a very interesting review, E. Satta et al. explored other aspects of the management of high-risk metabolic women, focusing on sexual dysfunctions in women with diabetic kidney disease and exploring the role of metabolic and hormonal variables and frequently used drugs on this important aspect impacting their quality of life.

Even though the papers published in this special issue are profoundly different in topic and study methodologies, they overall underline the importance of sex differences when examining CVD risk factors and their association with treatment, outcome, and mortality, especially in T2DM subjects.

*Giuseppina T. Russo* *Giuseppina T. Russo* *Giovannella Baggio* *Giovannella Baggio* *Maria Chiara Rossi* *Maria Chiara Rossi* *Alexandra Kautzky-Willer* *Alexandra Kautzky-Willer*
